# Editorial: Protein Misfolding and Proteostasis Impairment in Aging and Neurodegeneration: From Spreading Studies to Therapeutic Approaches

**DOI:** 10.3389/fnagi.2021.830779

**Published:** 2022-02-09

**Authors:** Claudia Duran-Aniotz, Ines Moreno-Gonzalez, Danilo B. Medinas, Rodrigo Morales

**Affiliations:** ^1^Latin American Institute for Brain Health (BrainLat), Universidad Adolfo Ibanez, Santiago, Chile; ^2^Center for Social and Cognitive Neuroscience, School of Psychology, Universidad Adolfo Ibanez, Santiago, Chile; ^3^Departamento Biologia Celular, Genetica y Fisiologia, Facultad de Ciencias, Instituto de Investigacion Biomedica de Malaga-IBIMA, Universidad de Malaga, Málaga, Spain; ^4^Networking Research Center on Neurodegenerative Diseases (CIBERNED), Madrid, Spain; ^5^Centro Integrativo de Biología y Química Aplicada, Universidad Bernardo O'Higgins, Santiago, Chile; ^6^Department of Neurology, The University of Texas Health Science Center at Houston, Houston, TX, United States; ^7^Biomedical Neuroscience Institute, Faculty of Medicine, University of Chile, Santiago, Chile; ^8^Center for Geroscience, Brain Health and Metabolism, Santiago, Chile; ^9^Program of Cellular and Molecular Biology, Center for Molecular Studies of the Cell, Institute of Biomedical Sciences, University of Chile, Santiago, Chile

**Keywords:** protein misfolding, proteostasis, aging, neurodegeneration, Alzheimer's disease

Misfolding, aggregation, and deposition of proteins in the nervous system are common features of several neurodegenerative diseases such as Alzheimer's disease (AD), Parkinson's disease (PD), Huntington's disease (HD), frontotemporal dementia (FTD), amyotrophic lateral sclerosis (ALS), and prion diseases, among others. Aging is the most relevant risk factor for all these diseases and recent evidence suggests that the buffering capacity of the proteostasis network decreases with aging and contributes to the etiology of these neuropathologies (Martinez et al., 2017). In this Research Topic, we have compiled a list of interesting manuscripts which describe protein misfolding and proteostasis impairment in aging and neurodegeneration.

AD is the most iconic disease associated with protein misfolding. This disease is the most common form of dementia in the elderly population, affecting about 10% of individuals over the age of 65, with its frequency increasing to nearly 40% by the age of 85. Pathologically, the most prominent features of AD are the extracellular deposition of amyloid-β (Aβ) peptides and the intracellular accumulation of hyper-phosphorylated tau (p-tau) proteins in the brain. These features have been associated with a series of detrimental events, including exacerbated brain inflammation and oxidative stress, leading to synaptic dysfunction and neuronal death. Recent data has emerged suggesting that the progressive axonal degeneration in early stages of AD is associated with Aβ and tau accumulation, which has been discussed by Salvadores et al.

Another pathological property of misfolded proteins involves their ability to spread or propagate within different anatomical structures of the nervous system through prion-like mechanisms (Gomez-Gutierrez and Morales, 2020). This has been particularly well-studied for Aβ and tau proteins involved in AD. Despite extensive research conducted in the last decade, the mechanisms dictating the organized spread and neurodegeneration associated with these disease-associated proteins is not completely understood. In this line, d'Errico and Meyer-Luehmann clearly summarized the current literature involving the spreading mechanisms of Aβ and tau pathology in AD. Their article focuses on evidence supporting the prion-like model for Aβ and tau spreading and how those proteins can be secreted and internalized by cells. They also highlight the need for better understanding of these disease pathways and the design of accurate and efficient intervention methods based on these mechanisms (d'Errico and Meyer-Luehmann).

A comprehensive review by McAlary et al. further describes evidence for the prion-like propagation of other proteins prone to misfold. Their article specifically focuses on amyotrophic lateral sclerosis (ALS), a fatal neurodegenerative disease affecting motoneurons and leading to death due to respiratory failure (Taylor et al., 2016). The authors cover literature involving biochemical and seeding properties of major ALS-linked proteins including superoxide dismutase 1 (SOD1), transactive response DNA-binding protein 43 (TDP-43) and fused in sarcoma (FUS) in different systems including invertebrate and mammalian models. TDP-43-positive inclusions are detected in over 90% of ALS patients and recent work has demonstrated its seeding potential. TDP-43 and FUS contain prion-like low complexity domains that can drive liquid-liquid phase separation forming membraneless organelles such as stress granules. These structures may link cellular stress responses to age-related proteostasis collapse, driving neurodegeneration. Collectively, the literature reviewed in this article supports the relevance of prion-like propagation of toxic protein species to ALS pathogenesis.

Knowledge collected on infectious prions has been used to explain relevant mechanisms associated with several neurodegenerative diseases. Importantly, prion diseases are unique among other neurodegenerative disorders due to their confirmed infectious etiology. Most mammals are susceptible to prion diseases either naturally or under experimental conditions. However, some animal species such as dogs, horses and rabbits have proven to be resistant to a diverse array of prion isolates. Several *in vitro* and *in vivo* studies have been conducted to identify the elements in the prion protein sequence that provides infection-resistance. These include the comparison of prion protein structures from animals susceptible and resistant to infection, *in vitro* prion conversion assays, and bioassays using actual animals and transgenic systems (mice and flies). Considering all these studies, few aminoacids in the prion protein (PrP) sequence have been identified as key to provide resistance to templated-conversion. This experimental evidence is conscientiously summarized and discussed by Myers et al. who hypothesize that these protective amino acids generate more compact and stable structures in the C-terminal domain of the prion protein, making it more resistant to acquire disease-associated isoforms. Research in this area is key to understanding the probability of certain animal species to get naturally infected by infectious prions and may help to develop drugs stabilizing specific domains involved in prion conversion.

Regarding mechanisms associated with ALS pathogenesis, the review by Jagaraj et al. systematically describes alterations of redox pathways in this disease. The authors present major pathways controlling redox balance, including sources of reactive oxygen species such as NADPH oxidases and defense antioxidant systems. The paper highlights the protein disulfide isomerases (PDIs), a class of oxidoreductases that promote disulfide bond formation in the endoplasmic reticulum (ER), as key components of the proteostasis network dysregulated in ALS. According to the evidence discussed, redox modifications such as S-nitrosylation and ALS-linked mutations in PDIs impair their redox activity, resulting in protein misfolding and disulfide-dependent aggregation. It will be of great importance determining whether such disulfide-crosslinked proteins can serve as templates to propagate toxic conformations along the central nervous system (CNS). The failure of previous clinical trials using redox-active compounds indicate that targeting redox mechanisms in ALS depends on more specific targets.

The study by Charif et al. uncovers important evidence involving TDP-43 toxicity. Under normal conditions, TDP-43 is localized in the nucleus. In ALS and other neurodegenerative diseases, however, TDP-43 is mislocalized to the cytoplasm where it may gain a toxic function (Taylor et al., 2016). Employing cell culture and transgenic mouse models, the authors show that cytoplasmic TDP-43 impairs the proteostasis network by inhibiting translation, which was monitored by puromycin labeling, in addition to preparation of mRNA-ribosome complexes from brain tissue. According to the authors, multiple mechanisms may be associated to suppression of protein synthesis by TDP-43 that deserve further investigation, including disruption of mRNA recruitment by ribosomes, sequestration of ribosome components into protein inclusions, aberrant formation of stress granules, ER stress and activation of the unfolded protein response, among others. Determination of the underlying pathogenic mechanisms triggered by cytoplasmic TDP-43 can lead to the design of novel therapeutics to restore translation rates in ALS.

As mentioned, a reduction in the capacity of homeostatic mechanisms during aging may increase the accumulation of misfolded proteins leading to age-related neurodegenerative diseases. One of the key pathways that mediates cell adaptation is the integrated stress response (ISR), which promotes translational arrest and induction of selected adaptive elements through the phosphorylation of the eukaryotic translation initiation factor 2 alpha (eIF2α). However, under chronic activation, ISR can also induce cell death, being considered an important regulator of neural physio(patho)logical conditions. With this focus, Martinez et al. (2021) reviewed evidence linking protein kinase R (PKR), an ISR sensor, to physiological conditions and neurodegenerative processes contributing to age-related pathologies. The PKR-eIF2α complex has been involved in both long-term potentiation (LTP) and long-term depression (LTD) in the hippocampus, physiological mechanisms that have been proposed to correlate with performance of mouse models in tasks of cognitive memory. Martinez et al. also described the role of the PKR signaling pathway in pathological conditions, including AD. Increased PKR levels have been identified in mouse and human samples, correlating with the severity of cognitive impairment. PKR involvement in other neurodegenerative diseases including PD and HD has also been widely documented (Martinez et al., 2021).

An emerging model explaining the impairment of cellular homeostasis during aging leading to subsequent development of neurodegenerative diseases involves mitochondrial dysfunction. The mitochondrial unfolded protein response (UPR^MT^), which involves the transcriptional activation of mitochondrial chaperones, proteases and antioxidant enzymes, is a mechanism to repair defective mitochondria relevant for neurological disorders. Muñoz-Carvajal and Sanhueza summarized several lines of investigation supporting this hypothesis, highlighting the factors that influence mitochondrial homeostasis during normal and pathological aging.

Similarly to ALS, spinal and bulbar muscular atrophy (SBMA) is classified as a motor neuron disorder (MND), with both diseases sharing common clinical manifestations and pathological features marked by accumulation of misfolded proteins in different areas of the nervous system involving multiple anatomical regions and cell types. On one hand, the different etiology of MNDs make the study of pathogenic mechanisms and search for therapeutic approaches challenging. On the other hand, both ALS and SBMA share a common pathological feature: the imbalance of the protein quality control system. The article by Cristofani et al. describe the pathological mechanisms involved in ALS and SBMA, specifically focused on the elements of the protein quality control system that are altered in these diseases. These include chaperones, protein degradation systems (the ubiquitin proteasome system and the autophagy-lysosomal pathway), the unfolded protein response, and the release of disease-associated proteins through extracellular vesicles. Along this line, the authors discuss how these different elements of the protein quality control system may be manipulated for therapeutic purposes.

Similarly, the review article by Troncoso-Escudero et al. discusses the different therapeutic approaches under development to treat the two most common movement disorders associated with misfolded proteins: PD and HD. Here, the authors focus on both preclinical approaches and clinical trials involving pharmacological, cellular replacement and genetic manipulation strategies, in addition to the use of growth factors and electrical modulation therapies. Pros and cons of each treatment avenue are thoroughly discussed, serving as a reference for clinicians and basic investigators.

Chaperones are relevant players in maintaining proper protein folding, facilitating that proteins acquire their final conformation. They are known as heat shock proteins (Hsp) as they are crucial in stress response. The activity of the molecular chaperones is compromised in chronic cellular stress and age-related neurodegenerative diseases, including AD, PD, and HD (Winklhofer et al., 2008). In fact, the expression of some Hsp is decreased in AD (Winklhofer et al., 2008). Overexpression of chaperones can decrease neurodegeneration, whereas their reduction may accelerate disease rate (Park et al., 2017). Tittelmeier et al. review the dual role of molecular chaperones in neurodegenerative diseases. On one hand, chaperones are necessary in maintaining cellular proteostasis by folding proteins and assisting degradation of those that are not properly folded. On the other hand, they can also mediate in the progression of several protein misfolding disorders, interfering in the aggregation and spreading of different disease-related amyloids. In addition, non-natively folded proteins can be degraded in lysosomes by chaperone-mediated autophagy (CMA). Chaperones associated with this specialized protein degradation machinery can become dysfunctional during brain aging, neurodegenerative disorders and even brain tumors. Auzmendi-Iriarte and Matheu (2021) review alterations observed in this autophagy mechanism in healthy and disease conditions and propose the development of CMA modulators to successfully intervene in different brain disorders. Chaperones not only assist in (re)folding of intracellular proteins, but they can also be secreted to the extracellular space. Alterations in secreted chaperones from neurons and glial cells have been lately associated with neurodegenerative disorders, as evaluated by Chaplot et al. This pathological mechanism may be key in amyloid spreading and a therapeutic target for prion-like disorders.

Overall, all the reports building this special issue further our understanding on the mechanisms contributing to protein misfolding processes, proteostasis impairment and neuronal dysfunction in aging and neurodegeneration. Considering normal and pathological aging as a multifactorial condition, a deep understanding of the cross-talk between protein misfolding, proteostasis and neurodegeneration could be useful to establish promising multi levels therapies.

## Author Contributions

CD-A, IM-G, DBM, and RM have contributed to manuscript writing and editing. CD-A coordinated, reviewed, and checked the final version. All authors have made a substantial intellectual contribution to this manuscript and approved it for publication.

## Funding

This work was funded by the following agencies: CD-A: Alzheimer's Disease Association 2018-AARG-591107, ANID/FONDEF ID20I10152, ANID/FONDECYT 1210622, and ANID/PIA/ANILLOS ACT210096. IM-G: 27565 2018 NARSAD, R21 AG067311-01 NIH, RYC-2017-21879 Ramon y Cajal and PID2019-107090RA-I00 Spanish Ministry of Science, B1-2019_06, and UMA20-FEDERJA-104. DBM: FONDECYT 1191538, Muscular Dystrophy Association 575897, and ALS Association 19-IIA-456. RM: Alzheimer's Association AARGD-18-566576, and NIH/NIA R01AI132695.

## Conflict of Interest

The authors declare that the research was conducted in the absence of any commercial or financial relationships that could be construed as a potential conflict of interest.

## Publisher's Note

All claims expressed in this article are solely those of the authors and do not necessarily represent those of their affiliated organizations, or those of the publisher, the editors and the reviewers. Any product that may be evaluated in this article, or claim that may be made by its manufacturer, is not guaranteed or endorsed by the publisher.
